# Balance or Strength? Reconsidering Muscle Metrics in Sagittal Malalignment in Adult Sagittal Deformity Patients

**DOI:** 10.3390/jcm14103293

**Published:** 2025-05-09

**Authors:** Donghua Huang, Zhan Wang, Mihir Dekhne, Atahan Durbas, Tejas Subramanian, Gabrielle Dykhouse, Robert N. Uzzo, Luis Felipe Colón, Stephane Owusu-Sarpong, Han Jo Kim, Francis Lovecchio

**Affiliations:** 1 Department of Orthopaedic Surgery, Hospital for Special Surgery, New York, NY 10021, USA; 2 Department of Orthopedic Surgery, The Second Affiliated Hospital Zhejiang University School of Medicine, Hangzhou 310009, China; 3 Weill Cornell Medicine, 1300 York Ave, New York, NY 10021, USA

**Keywords:** adult spinal deformity, sagittal alignment, trunk muscle balance

## Abstract

**Background/Objectives**: Atrophy of the paraspinal and psoas major muscles is closely linked to sagittal malalignment in adult spinal deformity (ASD). However, most studies overlook the balance between these muscle groups. This study investigates the relationship between trunk muscle balance and sagittal alignment in ASD patients. **Methods**: A single-institution database was reviewed for patients with sagittal malalignment (PT > 20° and PI–LL > 10°). Standard sagittal parameters were measured based on standing X-rays. The cross-section area (CSA) of trunk posterior muscles (CSA^P^: erector spinae and multifidus) and anterior muscles (CSA^A^: psoas) at L4 were measured based on a T2-weighted MRI. Patients with prior lateral fusions were excluded. Muscle balance was evaluated by the CSA ratio of trunk posterior to anterior muscles (CSA^P/A^). The relationship between sagittal alignment parameters and CSA^P^, CSA^A^, as well as CSA^P/A^ were analyzed using linear and quadratic regressions. Akaike information criteria (AIC) compared model fit. Subgroup analyses examined the relationship between sagittal alignment changes and different CSA^P/A^ levels. **Results**: A total of 112 patients met inclusion and exclusion criteria. CSA^P^ correlated linearly with SS (r^2^ = 0.057, *p* = 0.011), PT (r^2^ = 0.043, *p* = 0.028), and T4–L1PA mismatch (r^2^ = 0.044, *p* = 0.027). CSA^A^ showed no significant linear or quadratic relationships with sagittal spinal alignment parameters. In contrast, CSA^P/A^ was quadratically associated with LL (r^2^ = 0.056, *p* = 0.044), SS (r^2^ = 0.134, *p* < 0.001), PI (r^2^ = 0.096, *p* = 0.004), L1PA (r^2^ = 0.114, *p* = 0.001), and T4–L1PA mismatch (r^2^ = 0.094, *p* = 0.005). Quadratic models of CSA^P/A^ consistently had higher r^2^ and lower AIC values compared to the linear models of CSA^P^ for most sagittal alignment parameters, especially in SS, PI, L1PA, and T4–L1PA mismatch (AIC difference ≥4). Higher CSA^P/A^ is correlated to larger PI (and consequently, larger LL, SS, and L1PA). **Conclusions**: Trunk posterior–anterior muscle balance (CSA^P/A^) demonstrates a stronger relationship with sagittal alignment than individual muscle metrics. Quantitative MRI-based definitions of sarcopenia may need to be adjusted for PI.

## 1. Introduction

Adult spinal deformity (ASD) is a complex condition characterized by abnormal spinal alignment, often leading to significant disability, pain, and decreased quality of life [1,2,3,4]. In patients with ASD, a significant proportion experience sagittal imbalance, a critical parameter reflecting the spine’s failure to maintain proper upright stance in the sagittal plane [5,6]. Sagittal malalignment is a key driver of disability and pain [7,8,9].

The factors influencing sagittal balance in ASD are multifaceted, involving skeletal [10,11], neuromuscular [12], and compensatory mechanisms [13,14]. Among these, paraspinal muscles, specifically the posterior muscles (erector spinae and multifidus) [15,16,17,18,19,20] and anterior muscles (psoas) [19,20], have been closely linked to sagittal malalignment in ASD. However, existing research has focused solely on individual muscle metrics, neglecting the balance between trunk posterior and anterior muscles, which is vital for maintaining sagittal posture [21].

The purpose of this study aims to investigate the relationship between trunk posterior–anterior muscle balance and sagittal alignment in ASD patients. We hypothesize that the balance has a stronger relationship with sagittal alignment than individual muscle metrics. Moreover, the relationship between the ratio of trunk posterior to anterior muscles and sagittal alignment may be non-linear, potentially influencing alignment at varying rates and need to be adjusted for pelvic incidence (PI).

## 2. Materials and Methods

**Patient Enrollment**

Adult patients who underwent primary surgery for ASD from 2014 to 2023 and presented with sagittal malalignment, defined as a pelvic tilt (PT) > 20° and PI–lumbar lordosis (LL) > 10°, were included. Exclusion criteria were (1) under 18 years old; (2) lack of preoperative lumbar spine MRI or X-rays required for assessing spinal alignment or paravertebral muscles; (3) history of spinal procedures affecting the measurement of trunk muscles, such as lateral fusion with pedicle screw instrumentation; and (4) previous vertebral column infections.

**Data Collection and Radiographic Analysis**

Demographic data, including age, sex, BMI, smoking status, and medical comorbidities (diabetes, hypertension, osteoporosis, and dyslipidemia) were retrospectively obtained from electronic medical records.

**Spinal Alignment Assessment**

Spinal alignment was assessed using preoperative standing lateral and posteroanterior (PA) full-body X-rays. Standard spinopelvic parameters were measured as follows: (1) maximum coronal Cobb angle (Max Cobb); (2) thoracic kyphosis (TK; T4–T12); (3) LL (L1-S1); (4) sacral slope (SS); (5) PI; (6) PT; (7) sagittal vertical axis (SVA); (8) L1 pelvic angle (L1PA); (9) T4 pelvic angle (T4PA); (10) the L1PA offset (calculated as L1PA–normal L1PA, where normal L1PA was defined as 0.5 × PI − 21° [9]); and (11) the T4–L1PA mismatch (T4PA–L1PA).

**Trunk Muscle Assessment**

To study the relationship between muscles and spinal sagittal alignment, selecting the appropriate muscle group and evaluation method is essential. Trunk muscles [17,22], particularly the paralumbar muscles at the L4 level [23,24], are more relevant and reliable than extremity muscles for analyzing degenerative ASD with sagittal imbalance. Common evaluation methods include assessing muscle strength [16,17,18], quality (fat infiltration, FI) [4,25], and quantity (cross-sectional area, CSA) [26,27]. While muscle strength directly reflects function, it is less reliable due to poor reproducibility and variability in clinical testing [28,29,30]. On the contrary, muscle quality and quantity assessments, obtained via CT or MRI, offer reliable, objective, and consistent measures [30,31]. While fat infiltration reflects muscle quality, it poses challenges in directly quantifying posterior–anterior muscle balance. In contrast, CSA serves as a practical, reliable quantitative measure of overall muscle volume, enabling precise ratio-based analyses. The bilateral CSA of trunk posterior muscles (CSA^P^, including the erector spinae and multifidus) and anterior muscles (CSA^A^, the psoas) at the L4 level were measured based on a T2-weighted MRI. The primary comparison was between the CSA ratio of posterior–anterior muscle (CSA^P/A^) at the L4 level and sagittal alignment.

All spinal alignment and muscle measurements were performed using Sectra Workstation software (Model IDS7, Version 24.1, Linköping, Sweden), and two independent, blinded reviewers performed these assessments for each patient.

**Ethical Approval**

This study was conducted in accordance with the Declaration of Helsinki and approved by the local institutional review board (Approval No: 2018-1599). Due to its retrospective and observational design, the requirement for informed consent was waived.

**Statistical Analysis**

The Kolmogorov–Smirnov test was used to assess normality for samples larger than 50, while the Shapiro–Wilk test was applied for samples of 50 or fewer. Continuous data were reported as mean and standard deviation (SD) or median and interquartile range (IQR), based on their distribution.

The correlations between spinal alignment parameters and trunk muscles (CSA^P^, CSA^A^, and CSA^P/A^) were analyzed using simple linear and quadratic regressions, with scatter plots illustrating these relationships and fitted curves generated using GraphPad Prism 9.0. The model correlation coefficient (R^2^) and the Akaike information criterion (AIC) were calculated as a measure of fit for each model and subsequently compared across models. AIC = −2 × (Log-Likelihood) + 2k. k in the equation is the number of free parameters in the regression model. A higher R^2^ and a lower AIC value indicate a better model fit [32,33]. An AIC difference of ≥4 was regarded as a notable distinction between models [32].

Additionally, patients were stratified into three subgroups based on CSA^P/A^ values: low CSA^P/A^ (≤mean − 1/2 SD), middle CSA^P/A^ (mean ± 1/2 SD), and high CSA^P/A^ (≥mean + 1/2 SD). Differences in sagittal alignment parameters across these three subgroups were assessed using one-way analysis of variance (ANOVA) or the Kruskal–Wallis test, as appropriate, depending on the distribution of the data. All statistical analyses were performed using IBM SPSS Statistics (version 22.0; IBM Corp., New York, NY, USA), with a significance level set at *p*-value < 0.05.

## 3. Results

**Figure 1:**
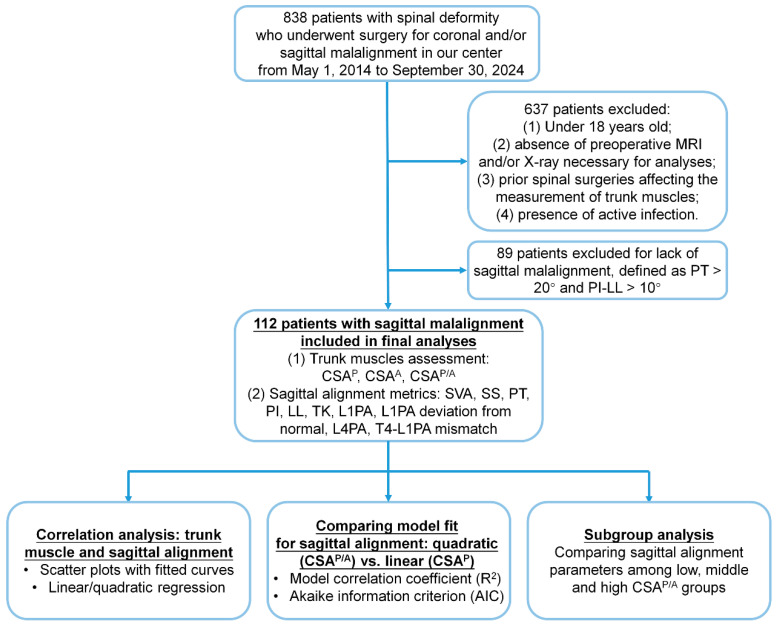
Flowchart of patient inclusion, exclusion, and analysis process.

A total of 112 patients (70.5% female) with sagittal ASD, average 66.3 ± 9.8 years of age, and BMI of 27.7 ± 6.0 kg/m^2^ met inclusion criteria and were included in the analysis (Figure 1). The patient demographics, radiographic measurements of spinal alignment, and CSA of trunk posterior and anterior muscles are reported in Table 1.

### 3.1. Simple Linear Regression Analysis of the Relationship Between Trunk Muscles and Sagittal Alignment

**Figure 2:**
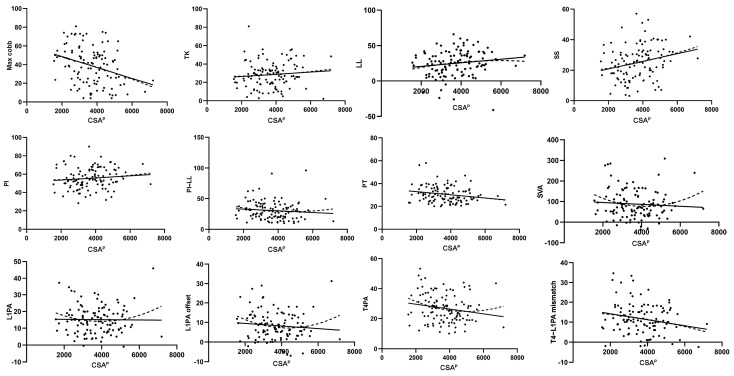
Scatter plot of the relationship between CSA^P^ and sagittal alignment parameters with linear and quadratic fit curves.

Linear regression analysis demonstrated significant associations between CSA^P^ and SS (r^2^ = 0.057, *p* = 0.011), PT (r^2^ = 0.043, *p* = 0.028), and the T4–L1PA mismatch (r^2^ = 0.044, *p* = 0.027) (Table 2). Scatter plots and fitted results (Figure 2) indicated that CSA^P^ exhibited linear relationships with most sagittal alignment parameters, including TK, LL, SS, PI, PI–LL, PT, T4PA, and T4–L1PA mismatch. In addition, CSA^P/A^ (Table 2) exhibited a significant relationship with LL (r^2^ = 0.042, *p* = 0.031) and SS (r^2^ = 0.057, *p* = 0.011). However, CSA^A^ (Table 2) showed no significant correlations with any sagittal alignment parameters. Notably, simple linear regression failed to fit the data for CSA^P/A^ with most sagittal alignment parameters, prompting further analysis using quadratic regression.

### 3.2. Quadratic Regression Analysis of the Relationship Between Trunk Muscles and Sagittal Alignment

**Figure 3:**
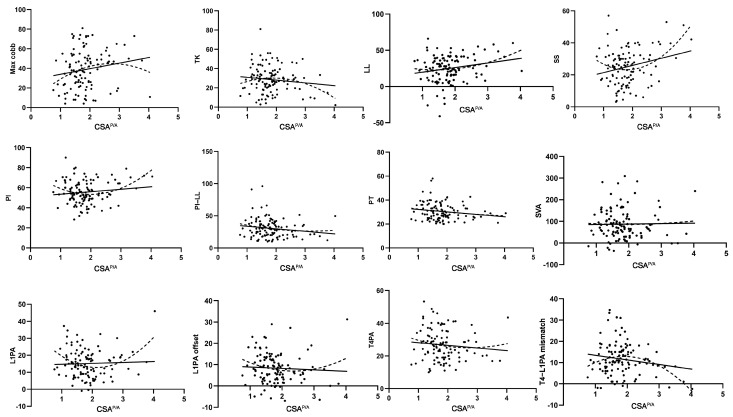
Scatter plot of the relationship between CSA^P/A^ and sagittal alignment parameters with linear and quadratic fit curves.

Quadratic regression analysis showed a significant association between CSA^P^ and SS (r^2^ = 0.058, *p* = 0.038) (Table 3). CSA^P/A^ exhibited significant relationships with multiple sagittal alignment parameters, including LL (r^2^ = 0.056, *p* = 0.044), SS (r^2^ = 0.134, *p* = 0.000), PI (r^2^ = 0.096, *p* = 0.004), L1PA (r^2^ = 0.114, *p* = 0.001), and the T4–L1PA mismatch (r^2^ = 0.094, *p* = 0.005) (Table 3). Scatter plot analysis and fitted results (Figure 3) also revealed that CSA^P/A^ exhibited quadratic relationships with most sagittal alignment parameters, particularly TK, LL, SS, PI, L1PA, L1PA offset, and T4–L1PA mismatch. Meanwhile, CSA^A^ showed no correlations with any sagittal alignment parameter (Table 3).

### 3.3. Comparison of Quadratic (CSA^P/A^) and Linear (CSA^P^) Models for Sagittal Alignment

On goodness-of-fit testing, the quadratic regression model (CSA^P/A^) demonstrated stronger relationships than the linear regression model (CSA^P^) for most sagittal spinal alignment parameters, with higher r^2^ values and lower AIC values (Table 4), including TK, LL, PI–LL, L1PA, L1PA offset, and T4–L1PA mismatch. Notably, the differences in AIC values between the two models exceeded four for SS (7.4), PI (8.0), L1PA (11.6), and T4–L1PA mismatch (4.1).

### 3.4. Subgroup Analyses of the Influence of CSA^P/A^ on Sagittal Alignment

**Figure 4:**
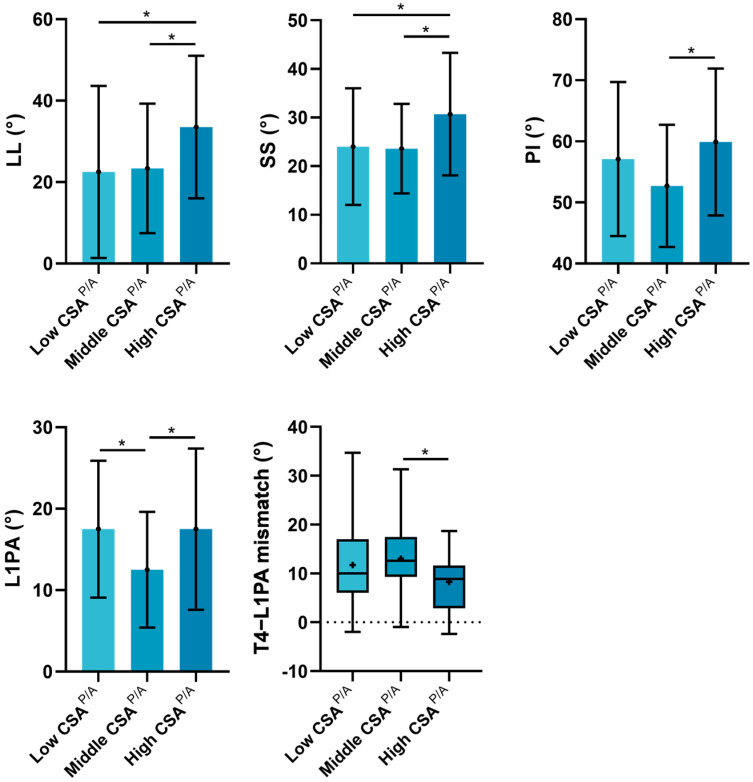
Comparison of sagittal alignment parameters (LL, SS, PI, L1PA, and T4–L1PA mismatch) among low, middle, and high CSA^P/A^ subgroups. *, *p* < 0.05 between the two groups.

Subgroup analyses (Table 5 and Figure 4) revealed significant differences among the three groups in SS (*p* = 0.030), PI (*p* = 0.031), L1PA (*p* = 0.007), and T4PA–L1PA (*p* = 0.041), with a trend of difference in LL (*p* = 0.056) and PT (*p* = 0.056). Additionally, pairwise comparisons indicated that the high CSA^P/A^ group exhibited significantly larger LL, SS, PI, and L1PA and smaller T4PA–L1PA compared to the middle and/or low CSA^P/A^ groups. However, when sagittal alignment was adjusted for PI, no significant difference was observed in PI–LL and L1PA offset among the three groups.

## 4. Discussion

Extensive research has demonstrated that trunk muscle quality is closely related to sagittal malalignment in ASD patients [15,16,17,18,19,20]. However, most studies only focus on muscle impairment alone, neglecting the balance between trunk posterior and anterior muscles. Although trunk posterior and anterior muscles are anatomically antagonistic, their functional interaction is synergistic, working together to maintain an upright posture and counteract gravity [21]. Thus, this study aimed to explore the relationship between trunk muscle balance and sagittal malalignment in patients with ASD. We identified a linear relationship between trunk posterior muscles (CSA^P^) and sagittal alignment, while the ratio of posterior to anterior muscles (CSA^P/A^) showed a quadratic association with alignment. Notably, the quadratic model consistently outperformed the linear model, with higher r^2^ values and lower AIC values, emphasizing the superior predictive value of posterior–anterior muscles balance over individual muscle metrics. Furthermore, a higher ratio of posterior to anterior muscles (higher CSA^P/A^) in the spine is correlated to larger PI (and consequently, larger LL, SS, and L1PA).

The current literature emphasizes the role of posterior muscles in maintaining sagittal alignment and functional mobility [18,34,35,36]. We also observed that trunk posterior muscles exhibited a linear relationship with most sagittal alignment parameters, whereas anterior muscles alone showed no significant association (Table 2 and Table 3, Figure 2). Notably, after further exploration, we identified a quadratic relationship between the ratio of posterior–anterior muscles and most sagittal parameters (Table 3 and Figure 3), and this association was markedly stronger than the linear relationship observed with posterior muscles alone (Table 4). This suggests that the balance between posterior and anterior muscles may be more important than either posterior or anterior muscles alone in maintaining sagittal stability and alignment in ASD patients. Such findings align with evidence from other populations, such as patients with low back pain [37] and male students [38], identifying a similar significance of this balance in influencing lumbar lordosis across these groups. Additionally, extensor and contractor muscle balance contributes to the stability of other joints, such as the knee [39,40], elbow, and shoulder [41,42], reducing the risk of injury. Taken as a whole, our findings suggest that future study of muscle metrics must evaluate individual (e.g., anterior and posterior) muscles and the balance between the two.

**Figure 5:**
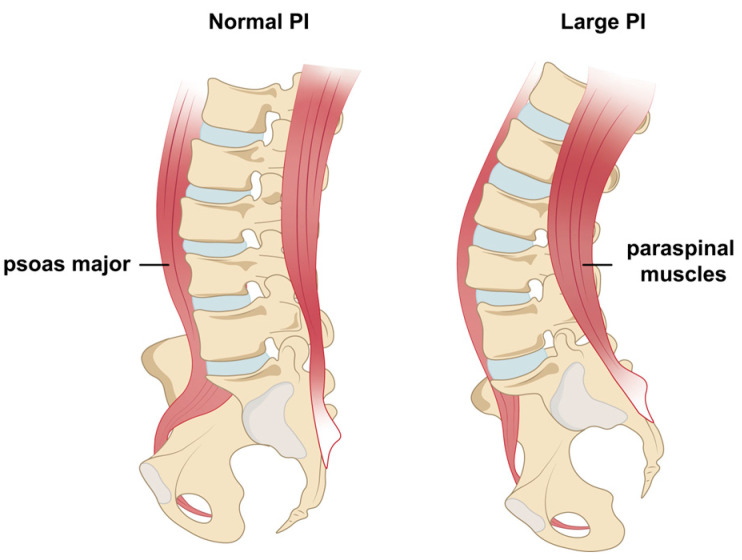
Illustration of asymmetric muscular morphological changes associated with large pelvic incidence (PI). A large PI typically results in increased lumbar lordosis (LL), which in turn leads to elongation and thinning of the psoas major on the convex side, reducing its cross-sectional area (CSA). Simultaneously, the paraspinal muscles on the posterior concave side become shortened and thickened, increasing their CSA. This asymmetric adaptation ultimately results in an elevated CSA ratio of the posterior concave paraspinal muscles to the anterior convex psoas major (CSA^P/A^).

Moreover, this quadratic association suggests a “U or inverted U-shaped” relationship between the ratio of posterior–anterior muscles and sagittal alignment parameters. We further explored the impact of different ranges of posterior–anterior-muscle ratio on sagittal alignment. Subgroup analysis revealed that a high posterior–anterior-muscle ratio is associated with significantly larger PI and consequently larger LL, SS, and L1PA (Table 5). A larger PI is usually accompanied by a deeper posterior concavity. It has been reported that muscles on the concave side may become shortened and stout with increased CSA, while those on the convex side may stretch, elongate, and exhibit a reduced CSA [43]. This phenomenon ultimately leads to an increased CSA ratio of the paraspinal muscles on the posterior concave side to the psoas major on the anterior convex side in cases of a large PI (Figure 5). Thus, in high-PI patients, using CSA alone may overestimate posterior muscle quantity and underestimate anterior muscle quality, a feature that could be problematic if one is studied in isolation [44,45]. To avoid these misinterpretations, MRI-based definitions of sarcopenia may need to be adjusted for PI. Furthermore, rehabilitation strategies could be personalized based on individual spinopelvic parameters. For instance, in patients with a large PI, posterior trunk muscles may exhibit compensatory hypertrophy due to structural adaptation rather than genuine functional strength. Therefore, for these high-PI patients, even when the CSA of posterior muscles appears not markedly reduced, targeted functional strengthening should remain a key focus in rehabilitation planning.

We also found that a high posterior–anterior-muscle ratio (posterior-dominant) is associated with smaller T4–L1PA mismatch (Table 5). A lower T4–L1PA mismatch has been associated with a decreased risk of mechanical failure [9]. Thus, posterior muscle dominance might be linked with better sagittal alignment compared to balanced or anterior-dominant muscle profiles. Such findings are supported by a prior study [35] that shows that trunk muscle ratios with posterior muscle predominance are associated with better physical performance and less back pain. However, a study on degenerative spondylolisthesis patients [46] presents opposing views: muscle trunk imbalance with predominance of posterior over anterior muscles was associated with functional disability. This discrepancy may originate from different outcome measures: we assessed sagittal alignment, while the opposing study used the Oswestry Disability Index (ODI) to evaluate functional disability. The ODI includes activities like lifting [47] and walking [48,49], which may bias results toward anterior muscle functions. When focusing on sagittal alignment in the present study, posterior muscle dominance means not only the inherent strength of the posterior muscles but also their dominant position in the synergistic interaction with the anterior muscles. This synergistic balance is crucial for maintaining sagittal alignment, as confirmed in a previous study [36]. Therefore, in rehabilitation exercises, focusing on strengthening trunk posterior muscles and maintaining posterior muscle dominance over anterior muscles may help reduce malalignment progression and enhance overall spinal stability in ASD patients. This may be a particularly effective strategy when employed in a “prehabilitation” manner [44].

There are limitations in the present study. First, due to the retrospective and observational design of this study, a causal relationship between muscle balance and spinal deformity cannot be inferred. This highlights the need for future prospective or interventional studies to confirm the hypotheses. In addition, although muscle characteristics are closely related to sagittal alignment, many other confounding factors (such as physical activity level, back pain, or prior spinal trauma) may also affect spinal alignment. However, these variables were not consistently available in our dataset, resulting in the failure to isolate their influence through multivariable analysis. Second, we predominantly focused on the key trunk posterior muscles (erector spinae and multifidus) and the key anterior muscle (psoas) when analyzing the relationship between posterior–anterior muscle balance and sagittal spinal malalignment. Most studies examining trunk muscle impairment in spine-related diseases have similarly focused mainly on these primary trunk posterior and/or anterior muscles [50,51,52]. However, other muscles also contribute to sagittal spinal stability, including additional posterior muscles such as the semispinalis [53] and quadratus lumborum [54] as well as anterior muscles like the rectus abdominis [55], external oblique [56], and transversus abdominis [57]. The CSAs of these muscles were not individually measured or included in our analysis due to the difficulty in accurately distinguishing and measuring them on MRI images [58] or because they were located outside the scanning range of lumbar MRI, which may limit the comprehensiveness of our findings. Future studies should expand the analysis to include additional muscle groups involved in trunk stabilization. Third, CSA does not capture muscle quality. While classifications such as the Goutallier classification have been used, there is no lumbar-specific measure of muscle quality. Future research should investigate more specific imaging modalities in the assessment of lumbar muscle quality [59]. Finally, although the paraspinal muscles at the L4 level have been shown to reliably reflect overall trunk muscle degeneration and stability [23,24], limiting measurements to this level may not fully capture muscle changes at higher deformity segments.

## 5. Conclusions

This study emphasizes the critical role of trunk muscle balance in sagittal alignment among ASD patients. We demonstrate that the posterior–anterior muscle balance, as captured by CSA^P/A^, correlates more strongly with sagittal alignment compared to individual muscle metrics. We encourage that future studies on lumbar musculature include this ratio as well as individual muscle parameters. Furthermore, the ratio between anterior and posterior muscles in the spine is correlated to PI (and consequently, LL and L1PA). Thus, MRI-based definitions of sarcopenia may need to be adjusted for PI.

## Figures and Tables

**Table 1:** Measurements of trunk posterior and anterior muscles, and spinal alignment for the 112 adult patients with sagittal spinal deformity.

Variable *	Mean (SD)
Age (year)	66.3 ± 9.8
Sex (M/F) (no.)	33:79
BMI (kg/m^2^) ^&^	27.7 ± 6.0
Smoker (current or former), n(%)	28(25.0%)
Diabetes, n(%)	5(4.5%)
Tumor, n(%)	13(11.6%)
Hypertension, n(%)	45(40.1%)
Osteoporosis, n(%)	20(17.9%)
Dyslipidemia, n(%)	32(28.6%)
Coronary artery disease, n(%)	11(9.8%)
Peripheral neuropathy, n(%)	1(0.9%)
Autoimmune, n(%)	4(3.6%)
Anxiety, n(%)	7(6.3%)
**Spinal alignment parameters**	
Max Cobb (°)	38.7 ± 19.9
TK (T4–T12) (°)	25.6 ± 13.9
LL (L1–S1) (°)	25.1 ± 18.5
SS (°)	25.1 ± 11.1
PI (°)	55.6 ± 11.6
PI–LL (°)	30.5 ± 15.1
PT (°)	30.5 ± 7.2
SVA (mm)	87.7 ± 69.8
L1PA (°)	15.1 ± 8.5
L1PA offset (°)	8.3 ± 7.3
T4PA (°)	26.8 ± 9.6
T4–L1PA mismatch (°)	11.6 ± 7.7
**Trunk muscle assessment ^#^**	
CSA^P^	3707.9 ± 1074.5
CSA^A^	2148.7 ± 718.4
CSA^P/A^	1.83 ± 0.59

Abbreviations: * Values are given as the mean and SD unless otherwise noted; SD, standard deviation; M, male; F, female; BMI, body mass index; ^&^, the data of four patients are missing; TK, thoracic kyphosis; LL, lumbar lordosis; SS, sacral slope; PI, pelvic incidence; PT, pelvic tilt; SVA, sagittal vertical axis; L1PA, L1 pelvic angle; L1PA offset, calculated as L1PA-(0.5 × PI − 21°); T4PA, T4 pelvic angle; T4–L1PA mismatch, calculated as T4PA–L1PA; ^#^, trunk muscles were evaluated at L4 level; CSA, the cross-sectional area; CSA^P^, CSA of posterior muscles (erector spinae and multifidus); CSA^A^, CSA of anterior muscles (psoas); and CSA^P/A^, the CSA ratio of trunk posterior to anterior muscles.

**Table 2:** Simple linear regression of the trunk muscles and radiological parameters of spinal alignment.

Assessment	Max Cobb	TK	LL	SS	PI	PI–LL	PT	SVA	L1PA	L1PA Offset	T4PA	T4–L1PA Mismatch
**CSA^P^**	**r^2^ = 0.100**	r^2^ = 0.009	r^2^ = 0.021	**r^2^ = 0.057**	r^2^ = 0.011	r^2^ = 0.009	**r^2^ = 0.043**	r^2^ = 0.005	r^2^ = 0.000	r^2^ = 0.010	r^2^ = 0.032	**r^2^ = 0.044**
	***p* = 0.001**	*p* = 0.329	*p* = 0.124	***p* = 0.011**	*p* = 0.266	*p* = 0.308	***p* = 0.028**	*p* = 0.464	*p* = 0.901	*p* = 0.301	*p* = 0.057	***p* = 0.027**
**CSA^A^**	**r^2^ = 0.210**	r^2^ = 0.024	r^2^ = 0.000	r^2^ = 0.004	r^2^ = 0.002	r^2^ = 0.003	r^2^ = 0.001	r^2^ = 0.011	r^2^ = 0.000	r^2^ = 0.001	r^2^ = 0.007	r^2^ = 0.011
	***p* = 0.000**	*p* = 0.101	*p* = 0.877	*p* = 0.502	*p* = 0.646	*p* = 0.590	*p* = 0.713	*p* = 0.268	*p* = 0.981	*p* = 0.735	*p* = 0.383	*p* = 0.270
**CSA^P/A^**	r^2^ = 0.029	r^2^ = 0.015	**r^2^ = 0.042**	**r^2^ = 0.057**	r^2^ = 0.016	r^2^ = 0.023	r^2^ = 0.029	r^2^ = 0.000	r^2^ = 0.001	r^2^ = 0.003	r^2^ = 0.010	r^2^ = 0.028
	*p* = 0.073	*p* = 0.196	***p* = 0.031**	***p* = 0.011**	*p* = 0.187	*p* = 0.108	*p* = 0.074	*p* = 0.859	*p* = 0.687	*p* = 0.563	*p* = 0.292	*p* = 0.080

Abbreviations: TK, thoracic kyphosis; LL, lumbar lordosis; SS, sacral slope; PI, pelvic incidence; PT, pelvic tilt; SVA, sagittal vertical axis; L1PA offset, calculated as L1PA-(0.5 × PI − 21°); T4PA, T4 pelvic angle; T4–L1PA mismatch, calculated as T4PA–L1PA; CSA, the cross-sectional area; CSA^P^, CSA of posterior muscles (erector spinae and multifidus); CSA^A^, CSA of anterior muscles (psoas); CSA^P/A^, the CSA ratio of trunk posterior to anterior muscles. The correlations with significance (*p* < 0.05) are highlighted with bold; The darker the red background, the stronger the correlation (the higher r² value).

**Table 3:** Quadratic regression of the trunk muscles and radiological parameters of spinal alignment.

Assessment	Max Cobb	TK	LL	SS	PI	PI–LL	PT	SVA	L1PA	L1PA Offset	T4PA	T4–L1PA Mismatch
**CSA^P^**	**r^2^ = 0.100**	r^2^ = 0.009	r^2^ = 0.024	**r^2^ = 0.058**	r^2^ = 0.012	r^2^ = 0.016	r^2^ = 0.043	r^2^ = 0.042	r^2^ = 0.029	r^2^ = 0.042	r^2^ = 0.048	r^2^ = 0.045
	***p* = 0.003**	*p* = 0.608	*p* = 0.261	***p* = 0.038**	*p* = 0.525	*p* = 0.404	*p* = 0.089	*p* = 0.096	*p* = 0.202	*p* = 0.096	*p* = 0.068	*p* = 0.083
**CSA^A^**	**r^2^ = 0.212**	r^2^ = 0.024	r^2^ = 0.000	r^2^ = 0.005	r^2^ = 0.003	r^2^ = 0.005	r^2^ = 0.001	r^2^ = 0.013	r^2^ = 0.009	r^2^ = 0.007	r^2^ = 0.008	r^2^ = 0.016
	***p* = 0.000**	*p* = 0.259	*p* = 0.974	*p* = 0.762	*p* = 0.841	*p* = 0.768	*p* = 0.931	*p* = 0.481	*p* = 0.623	*p* = 0.664	*p* = 0.658	*p* = 0.423
**CSA^P/A^**	r^2^ = 0.050	r^2^ = 0.048	**r^2^ = 0.056**	**r^2^ = 0.134**	**r^2^ = 0.096**	r^2^ = 0.028	r^2^ = 0.029	r^2^ = 0.001	**r^2^ = 0.114**	r^2^ = 0.031	r^2^ = 0.018	**r^2^ = 0.094**
	*p* = 0.060	*p* = 0.070	***p* = 0.044**	***p* = 0.000**	***p* = 0.004**	*p* = 0.209	*p* = 0.196	*p* = 0.945	***p* = 0.001**	*p* = 0.184	*p* = 0.376	***p* = 0.005**

Abbreviations: TK, thoracic kyphosis; LL, lumbar lordosis; SS, sacral slope; PI, pelvic incidence; PT, pelvic tilt; SVA, sagittal vertical axis; L1PA offset, calculated as L1PA-(0.5 × PI − 21°); T4PA, T4 pelvic angle; T4–L1PA mismatch, calculated as T4PA–L1PA; CSA, the cross-sectional area; CSA^P^, CSA of posterior muscles (erector spinae and multifidus); CSA^A^, CSA of anterior muscles (psoas); CSA^P/A^, the CSA ratio of trunk posterior to anterior muscles. The correlations with significance (*p* < 0.05) are highlighted with bold; The darker the red background, the stronger the correlation (the higher r² value).

**Table 4:** Comparison of AIC values between the linear model (CSA^P^) and quadratic model (CSA^P/A^) in relation to spinal alignment parameters.

Regression Model	Muscle Parameter	Max Cobb	TK	LL	SS	PI	PI–LL	PT	SVA	L1PA	L1PA Offset	T4PA	T4–L1PA Mismatch
Linear	CSA^P^	867.4	798.0	859.5	742.3	756.2	816.5	646.3	1159.3	687.2	651.6	711.0	662.4
Quadratic	CSA^P/A^	875.4	795.5	857.5	734.8	748.1	816.3	649.9	1161.7	675.6	651.2	714.7	658.4
Decreased AIC value from linear to quadratic model		−8.0	2.5	2.0	7.4	8.0	0.2	−3.6	−2.4	11.6	0.4	−3.7	4.1

Abbreviations: AIC, Akaike information criterion; TK, thoracic kyphosis; LL, lumbar lordosis; SS, sacral slope; PI, pelvic incidence; PT, pelvic tilt; SVA, sagittal vertical axis; L1PA offset, calculated as L1PA-(0.5 × PI − 21°); T4PA, L4 pelvic angle; T4–L1PA mismatch, calculated as T4PA–L1PA; CSA^P^, CSA of posterior muscles (erector spinae and multifidus); CSA^P/A^, the CSA ratio of trunk posterior to anterior muscles.

**Table 5:** Comparisons of spinal alignment parameters in subdividing three CSA^P/A^ groups.

Parameters	CSA^P/A^			*p*-Value			
	Low Group (*n* = 38)	Middle Group (*n* = 52)	High Group (*n* = 22)	Total	L Versus M	L Versus H	M Versus H
Max Cobb (°)	36.3 (17.2, 49.3)	36.5 (25.3, 52.5)	47.5 (34.2, 55.0)	0.207 ^$^	NS	NS	NS
TK (°) ^#^	30.8 ± 15.9	27.8 ± 12.7	26.6 ± 13.2	0.459 *	NS	NS	NS
LL (°)	22.5 ± 21.1	23.4 ± 15.9	33.5 ± 17.5	0.056 *	NS	**0.026**	**0.031**
SS (°)	24.0 ± 12.0	23.6 ± 9.2	30.7 ± 12.6	0.030 *	NS	**0.023**	**0.012**
PI (°)	57.1 ± 12.6	52.7 ± 10.0	59.9 ± 12.0	0.031 *	NS	NS	**0.015**
PI–LL (°)	30.7 (21.5, 41.9)	29.4 (19.9, 37.8)	21.4 (17.6, 34.3)	0.226 ^$^	NS	NS	NS
PT (°) ^#^	31.0 (26.1, 38.6)	27.7 (24.9, 34.0)	29.0 (25.2, 32.9)	0.056 ^$^	NS	NS	NS
SVA (mm) ^#^	85.5 (48.9, 136.7)	70.4 (36.7, 112.6)	66.2 (34.1, 137.1)	0.790 ^$^	NS	NS	NS
L1PA (°)	17.5 ± 8.4	12.5 ± 7.1	17.5 ± 9.9	0.007 *	**0.005**	NS	**0.018**
L1PA offset (°) ^#^	9.6 (3.9, 16.0)	6.5 (3.0, 11.8)	6.4 (3.2, 10.5)	0.195 ^$^	NS	NS	NS
T4PA (°) ^#^	28.9 (22.0, 36.3)	24.1 (18.9, 31.3)	23.3 (18.6, 30.6)	0.147 ^$^	NS	NS	NS
T4–L1PA mismatch (°) ^#^	10.0 (6.0, 17.0)	12.6 (9.3, 17.5)	8.9 (2.9, 11.7)	**0.041 ^$^**	NS	NS	**0.040**

Abbreviations: Mean and standard deviation is shown except for ^#^, where median and interquartile range (Q1–Q3) are given; CSA, the cross-sectional area; CSA^P/A^, the CSA ratio of trunk posterior to anterior muscles; TK, thoracic kyphosis; LL, lumbar lordosis; SS, sacral slope; PI, pelvic incidence; PT, pelvic tilt; SVA, sagittal vertical axis; L1PA offset, calculated as L1PA-(0.5 × PI − 21°); T4PA, L4 pelvic angle; T4–L1PA mismatch, calculated as T4PA–L1PA; L, low group; M, middle group; H, high group; *, a one-way analysis of variance (ANOVA); ^$^, Kruskal–Wallis test; and NS, no significance. Significances (*p* < 0.05) are highlighted with bold.

## Data Availability

The data presented in this study are available on request from the corresponding author due to institutional policies and patient confidentiality regulations. Data sharing is restricted to protect sensitive patient information in compliance with ethical guidelines and IRB-approved protocols.

## Abbreviations

The following abbreviations are used in this manuscript:

AIC	Akaike information criterion
ASD	Adult spinal deformity
BMI	Body mass index
CSA	Cross-sectional area
CSAA	Cross-sectional area of anterior muscles
CSAP	Cross-sectional area of posterior muscles
CSAP/A	Cross-sectional area ratio of posterior to anterior muscles
CT	Computed tomography
FI	Fat infiltration
IQR	Interquartile range
LL	Lumbar lordosis
L1PA	L1 pelvic angle
MRI	Magnetic resonance imaging
NS	No significance
PA	Posteroanterior
PI	Pelvic incidence
PT	Pelvic tilt
SD	Standard deviation
SS	Sacral slope
SVA	Sagittal vertical axis
T4PA	T4 pelvic angle
TK	Thoracic kyphosis
